# Mendelian randomization of plasma lipidome and temporomandibular joint disorders

**DOI:** 10.1097/MD.0000000000050984

**Published:** 2026-10-16

**Authors:** Ye Jiang, Huiqi Yuan, Yongping Cao

**Affiliations:** a Department of Orthopedics, Peking University First Hospital, Beijing, China; b Department of Public Health Administration, School of Public Health, Capital Medical University, Beijing, China.

**Keywords:** lipids, Mendelian randomization, Temporomandibular joint disorder

## Abstract

Mendelian randomization (MR) was used to investigate the possible causal association between lipids (LP) and temporomandibular joint disorders (TMD). To investigate the causal relationship between circulating LP and TMD, we conducted a 2-sample MR analysis. Exposure data comprised summary statistics for 179 LP from the GeneRISK genome-wide association study (N = 7174 samples). Outcome data were derived from the FinnGen genome-wide association study for TMD (N = 185169 samples). Primary causal estimates were generated using inverse-variance weighted regression, with sensitivity analyses including MR-Egger, weighted median, and Mendelian Randomization Pleiotropy RESidual Sum and Outlier. Robustness was assessed through pleiotropy-robust methods, heterogeneity testing, and leave-one-out analysis. Our analysis identified specific lipid species associated with temporomandibular joint disorder risk. Diacylglycerol (18:1_18:1) (odds ratio [OR] = 1.067, 95% confidence interval [CI] = 1.016−1.121, *P* = .010) and several triacylglycerols (TGs) were significantly associated with increased risk: TG (50:3) (OR = 1.053, 95% CI = 1.006–1.102, *P* = .028), TG (52:3) (OR = 1.073, 95% CI = 1.031–1.118, *P* = .001), TG (53:3) (OR = 1.062, 95% CI = 1.017–1.109, *P* = .006), TG (56:5) (OR = 1.057, 95% CI = 1.005–1.111, *P* = .032), and TG (56:6) (OR = 1.066, 95% CI = 1.020–1.114, *P* = .004). In contrast, Phosphatidylcholine (15:0_18:2) (OR = 0.953, 95% CI = 0.920–0.987, *P* = .007) and Phosphatidylcholine (16:0_18:2) (OR = 0.961, 95% CI = 0.928–0.996, *P* = .031) were associated with a reduced risk. Our MR analysis provides evidence supporting a putative causal relationship between specific plasma lipid species and the development of TMD. These findings highlight the potential role of lipid metabolism in TMD pathogenesis and warrant further investigation.

## 1. Introduction

temporomandibular disorder(s) (TMD) is a collection of conditions affecting the temporomandibular joint (TMJ), masticatory muscles, and other tissues. As the second most prevalent musculoskeletal disorder globally, it induces oral and facial discomfort and deformity, primarily characterized by face and preauricular pain, restricted jaw mobility, and joint crepitus.^[1]^ The worldwide frequency of the condition is around 34%, often linked to negative consequences such as compromised health, depression, and sleep disturbances, which significantly diminish patients’ quality of life.^[2,3]^ The particular manifestations of TMD include degenerative joint diseases, muscular disorders, and osteoarthritis, with the most prevalent forms now being pain-related and joint illnesses.^[4,5]^ Symptoms associated with pain encompass face discomfort and headaches, joint manifestations such as subluxation, limited mouth opening, and degenerative joint disease.^[6–8]^

While management ranges from conservative approaches (e.g., physiotherapy, occlusal splints, nonsteroidal anti-inflammatory drugs) to surgical intervention for refractory cases,^[9–11]^ understanding underlying pathophysiological mechanisms remains key to developing targeted therapies. Lipid metabolism dysregulation is a recognized contributor to inflammation and nociception.^[12,13]^ Pro-inflammatory lipids (LP) (e.g., diacylglycerols (DAG), triglycerides - TG) can activate signaling pathways (e.g., nuclear factor kappa B) driving synovitis and tissue degradation,^[14,15]^ while phospholipids (e.g., phosphatidylcholines [PC]) are vital for membrane integrity and cellular signaling in neural and articular tissues.^[16,17]^ Altered lipid profiles have been observed in synovial fluid and systemically in other joint disorders like osteoarthritis, suggesting a potential role in TMD pathogenesis. However, establishing a causal relationship between specific circulating lipid species and TMD risk is challenging due to confounding factors (e.g., stress, diet, comorbidities) and potential reverse causation in observational studies.^[18–20]^

Lipidomics enables comprehensive profiling of lipid species. Applying lipidomics within a Mendelian randomization (MR) framework offers a powerful approach to strengthen causal inference. MR utilizes genetic variants, primarily single nucleotide polymorphisms (SNPs), as instrumental variables (IVs). These IVs, fixed at conception and largely independent of confounding environmental factors and disease progression, allow for more robust assessment of the potential causal effect of an exposure (specific LPs) on an outcome (TMD) by mitigating biases inherent in observational designs.^[21]^

Critically, the precise causal role of the plasma lipidome in TMD etiology remains unclear, and no prior study has employed MR to investigate this relationship. Therefore, this study aimed to perform a comprehensive 2-sample MR analysis to evaluate the potential causal effects of a wide array of genetically predicted plasma lipid species on the risk of developing TMD. This study is the first to use MR to investigate the causal relationship between lipid levels and TMD.

## 2. Materials and methods

### 2.1. Study design and core assumptions

This 2-sample MR study was designed based on 3 foundational assumptions: Relevance: All selected genetic instruments (SNPs) are strongly associated with plasma lipid levels. Instrument strength was validated quantitatively via F-statistics, with all instruments satisfying *F* > 10. Detailed F-statistics for each SNP are reported in Table S1, Supplemental Digital Content 1.

This 2-sample MR study was designed based on 3 core assumptions. First, relevance requires that the selected genetic instruments (SNPs) are sufficiently associated with the exposure. Instrument strength was evaluated using *F*-statistics, and all retained instruments had *F*-statistics >10; detailed values are provided in Supplementary Table S1, Supplemental Digital Content 1. Second, the independence assumption requires that the genetic instruments are not associated with potential confounders of the lipid–TMD relationship. Although this assumption cannot be directly verified using summary-level data, potential violations due to horizontal pleiotropy were explored using the MR-Egger intercept and Mendelian Randomization Pleiotropy RESidual Sum and Outlier (MR-PRESSO) global test. Third, the exclusion restriction assumption requires that the genetic instruments affect TMD primarily through the lipid exposure rather than through alternative pathways. Potential violations were investigated using heterogeneity tests, MR-Egger regression, MR-PRESSO, and leave-one-out (LOO) analyses.

### 2.2. Statement of ethics

In this work, a publicly available genome-wide association study (GWAS) dataset was used. It was not necessary to get any new ethical approval for the research since it contained an analysis of already collected summary-level data.

### 2.3. Data sources and population characteristics

Publicly available GWAS summary statistics were used in this study, eliminating the need for new ethical approvals. No individual-level data were accessed. Exposure Data: Plasma lipid levels were obtained from the GeneRISKcohort,^[22]^ comprising 7174 Finnish individuals of European ancestry. A total of 179 lipid species across 13 lipid classes were measured using high-resolution shotgun lipidomics. Measurement reliability was supported by previous reports of high intra-assay reproducibility (coefficient of variation <10%) and cross-validation with targeted mass spectrometry.^[23,24]^ Participant mean age was 52.6 ± 11.3 years, with a female-to-male ratio of 1.1:1. Outcome Data: GWAS summary statistics for TMD were sourced from the FinnGen^[25]^ release 9 datasets (https://results.finngen.fi/en), including 18,517 diagnosed cases and 185,169 controls. TMD diagnoses were based on International Classification of Diseases, 10th Revision code K07.6, confirmed via national health registries with physician validation. The cohort was ethnically homogeneous (Northern European), minimizing population stratification. Mean age was 55.2 ± 13.8 years; females comprised 62% of TMD cases.

### 2.4. Genetic instrument selection

To obtain a sufficient number of genetic instruments for lipid species that lacked variants reaching conventional genome-wide significance, SNPs significantly associated with lipid levels at *P* < 5 × 10^−6^ were initially selected. This threshold is less stringent than the conventional genome-wide significance threshold of *P* < 5 × 10^−8^ and was adopted to increase the number of available instruments and statistical power. However, the relaxed threshold may also increase the potential for weak-instrument bias and false-positive findings. To mitigate this concern, linkage disequilibrium clumping was performed using *r*^*2*^ < 0.001 and a physical distance threshold of >10,000 kb, and instrument strength was evaluated using *F*-statistics. Only SNPs with *F* > 10 were retained. The smallest *F*-statistic in our study was 19.248, and detailed *F*-statistics are provided in Supplementary Table S1, Supplemental Digital Content 1.

### 2.5. MR analysis

The primary analysis employed inverse variance weighting (IVW), assuming no horizontal pleiotropy and homogeneous SNP effects. To test the robustness of causal estimates, we applied additional MR methods with different assumptions: MR-Egger Regression: Accounts for directional pleiotropy; non-zero intercept indicates violation of exclusion restriction. Weighted Median (WM): Yields consistent estimates even if up to 50% of instruments are invalid. Simple and Weighted Mode: Based on clustering instruments with similar causal estimates. Bayesian Weighted Mendelian Randomization (BWMR): Incorporates a variational expectation-maximization algorithm to model pleiotropy and weak instrument uncertainty. This method provides outlier detection and bias correction under polygenic assumptions. Results across methods were compared to assess consistency; discrepancies were explored in sensitivity analyses.

### 2.6. Sensitivity and pleiotropy analyses

Heterogeneity: Cochran’s *Q* test was applied to IVW and MR-Egger models to assess heterogeneity across SNPs. Horizontal Pleiotropy: MR-Egger intercept and MR-PRESSO global test identified directional and outlier pleiotropy. MR-PRESSO distortion test assessed the impact of outlier removal. LOO Analysis: Each SNP was iteratively removed to detect influential instruments. (Supplementary Table S2, Supplemental Digital Content 2)

### 2.7. Reverse MR and bidirectional testing

For lipid species showing significant causal associations with TMD in the primary MR analysis, reverse MR analysis was conducted to investigate the possibility of reverse causality. Genetic instruments for TMD were selected at *P* < 5 × 10^−6^ and subjected to linkage disequilibrium clumping using *r*^*2*^ < 0.001 and a physical distance threshold of >10,000 kb. The same MR approaches, including IVW, MR-Egger, and WM, were applied. The number of SNPs and corresponding F-statistics are reported in Supplementary Table S3, Supplemental Digital Content 3. Results of the reverse MR analyses are presented in Figure 1, together with the corresponding effect estimates and confidence interval (CIs).

**Figure 1.**
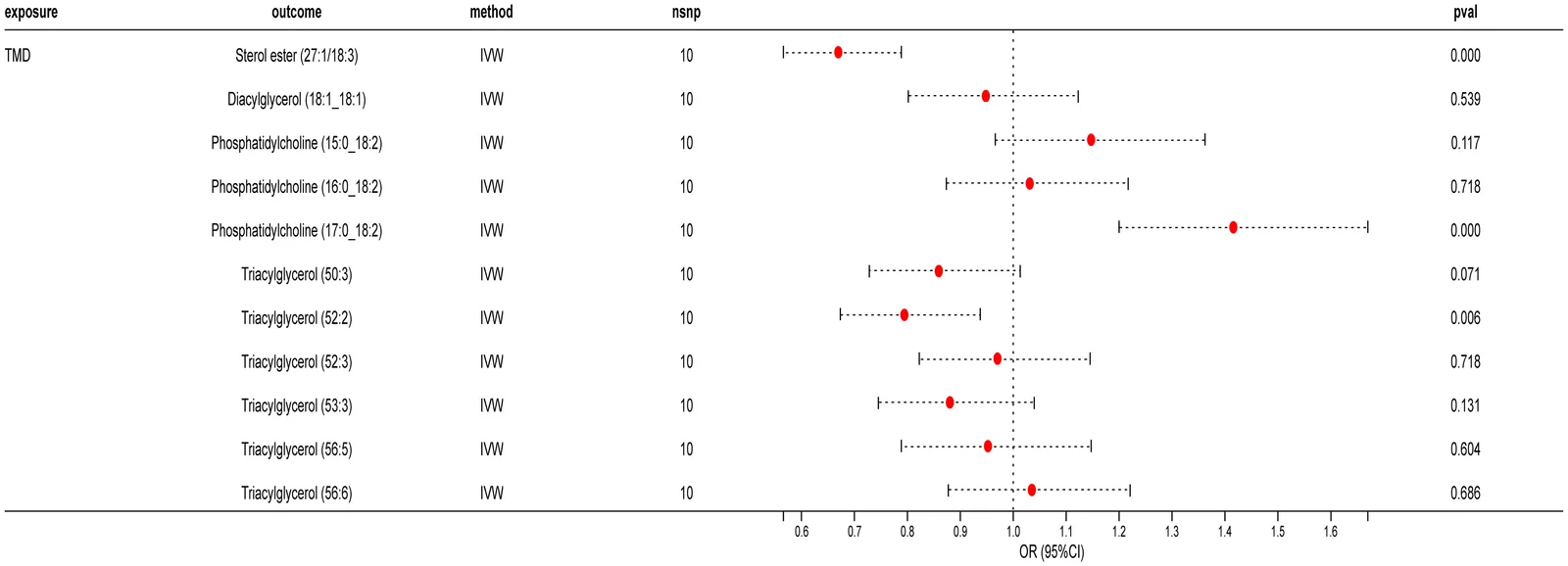
Mendelian randomization estimation for causality between revision of TMD and lipidomes. TMD = temporomandibular joint disorders.

### 2.8. Statistical power and software

All analyses were performed using R (v4.4.1) with the TwoSampleMR^[26]^ and MendelianRandomization^[27]^ packages. Analysis scripts and summary data processing pipelines are available on GitHub, ensuring transparency and reproducibility per Strengthening the Reporting of Observational Studies in Epidemiology using Mendelian Randomization recommendations.

## 3. Results

### 3.1. Causal connection between plasma lipidome and TMD

The causal impact of plasma lipid profiles on TMD was evaluated using 2-sample MR (Fig. 2) We used IVW as the main approach and utilized 4 other MR analysis techniques as supplementary methods (all supplemental content of this article). Their direction must align with the IVW findings, significantly enhancing the dependability of causation. Figure 3 illustrates that, based on the IVW method, Sterol ester (27:1/14:0, 27:1/18:3), DAG (18:1_18:1), Phosphatidylinositol (18:0_20:4), and TG (50:3, 52:2, 52:3, 53:3, 54:3, 56:5, 56:6, 58:7) are identified as risk factors for TMD, while PC (15:0_18:2, 16:0_16:0, 16:0_18:2, 17:0_18:2) serves as a protective factor (Table 1).

**Table 1:** The Mendelian Randomization analysis of the results for the association between plasma lipidome(exposure) and TMD (outcome).

Outcome	Exposure	Method	NSNP	Or	or_lci95	or_uci95	*P* val
Temporomandibular joint disorders	Diacylglycerol (18:1_18:1) levels	MR Egger	23	1.070	0.950	1.206	.276
		Weighted median	23	1.059	0.988	1.134	.108
		Inverse variance weighted	23	1.067	1.016	1.121	.010
		Simple mode	23	1.042	0.927	1.173	.497
		Weighted mode	23	1.084	0.977	1.202	.144
Temporomandibular joint disorders	Phosphatidylcholine (15:0_18:2) levels	MR Egger	39	0.934	0.868	1.004	.073
		Weighted median	39	0.953	0.898	1.012	.117
		Inverse variance weighted	39	0.953	0.920	0.987	.007
		Simple mode	39	0.991	0.893	1.099	.859
		Weighted mode	39	0.958	0.899	1.022	.203
Temporomandibular joint disorders	Phosphatidylcholine (16:0_18:2) levels	MR Egger	36	0.934	0.866	1.009	.092
		Weighted median	36	0.950	0.896	1.007	.087
		Inverse variance weighted	36	0.962	0.928	0.996	.031
		Simple mode	36	0.960	0.859	1.074	.483
		Weighted mode	36	0.952	0.898	1.010	.110
Temporomandibular joint disorders	Triacylglycerol (50:3) levels	MR Egger	30	1.008	0.905	1.123	.881
		Weighted median	30	1.075	1.007	1.147	.031
		Inverse variance weighted	30	1.053	1.006	1.102	.028
		Simple mode	30	1.132	0.986	1.300	.090
		Weighted mode	30	1.119	1.000	1.252	.060
Temporomandibular joint disorders	Triacylglycerol (52:3) levels	MR Egger	30	1.043	0.943	1.154	.417
		Weighted median	30	1.080	1.012	1.153	.020
		Inverse variance weighted	30	1.062	1.017	1.109	.006
		Simple mode	30	1.079	0.957	1.216	.226
		Weighted mode	30	1.091	1.005	1.185	.047
Temporomandibular joint disorders	Triacylglycerol (53:3) levels	MR Egger	25	1.115	0.982	1.266	.106
		Weighted median	25	1.073	1.002	1.148	.043
		Inverse variance weighted	25	1.057	1.005	1.111	.032
		Simple mode	25	1.076	0.936	1.235	.313
		Weighted mode	25	1.090	0.989	1.203	.096
Temporomandibular joint disorders	Triacylglycerol (56:5) levels	MR Egger	24	1.115	0.988	1.258	.091
		Weighted median	24	1.065	1.002	1.131	.043
		Inverse variance weighted	24	1.066	1.020	1.114	.004
		Simple mode	24	1.008	0.896	1.133	.897
		Weighted mode	24	1.013	0.911	1.126	.816
Temporomandibular joint disorders	Triacylglycerol (56:6) levels	MR Egger	27	1.075	0.978	1.181	.147
		Weighted median	27	1.066	1.003	1.134	.040
		Inverse variance weighted	27	1.074	1.031	1.118	.001
		Simple mode	27	1.066	0.959	1.185	.247
		Weighted mode	27	1.067	0.990	1.151	.100

**Figure 2.**
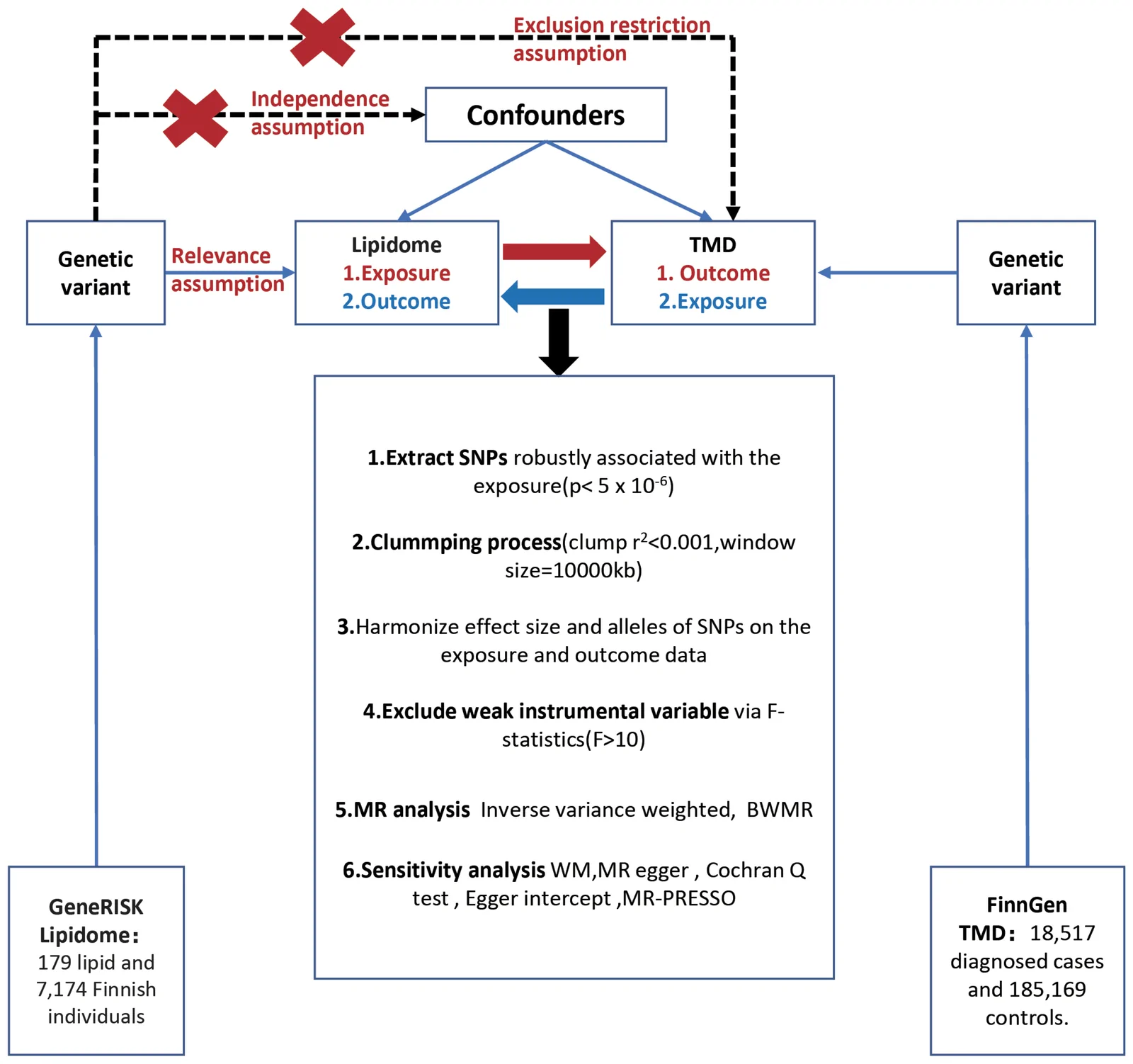
Flowchart of the MR study design. CI = confidence interval, OR = odds ratio, MR = Mendelian randomization.

**Figure 3.**
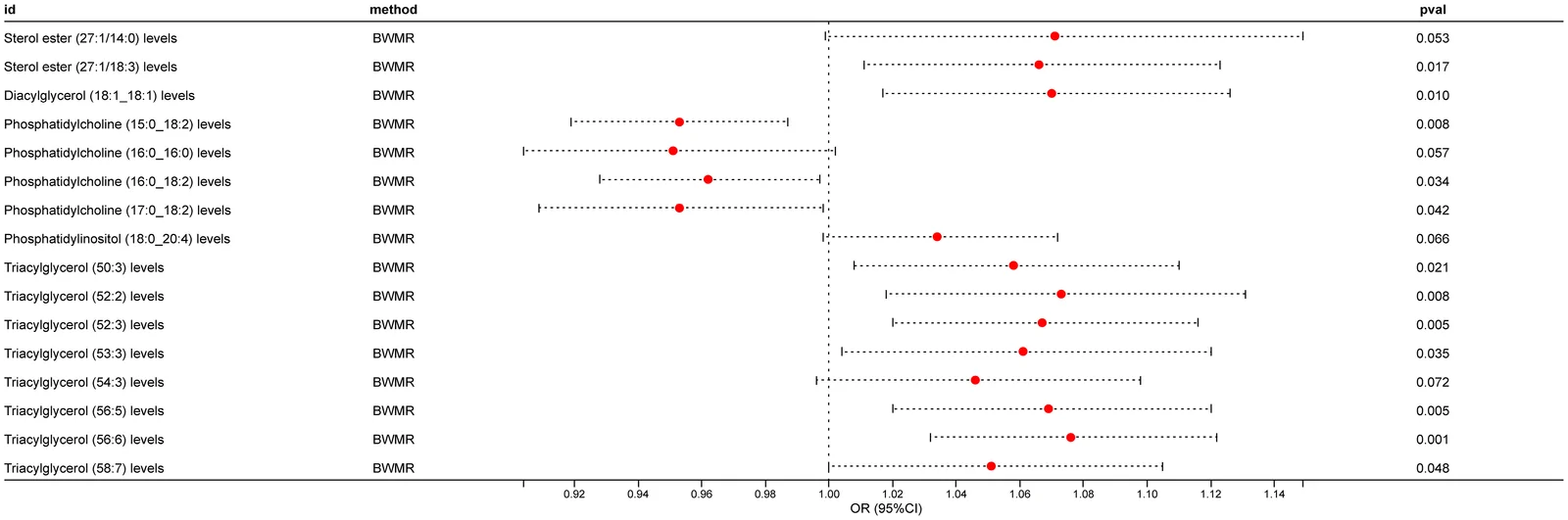
Forest plot of results of Bayesian weighted Mendelian randomization analysis of lipidomes in response to TMD. TMD = temporomandibular disorder(s).

Subsequently, we corroborated this finding by Bayesian weighted MR, as seen in Figure 4, identifying Sterol ester (27:1/18:3), DAG (18:1_18:1), TG (50:3), and Sterol Glycerol (27:1/18:3). TG (52:2), TG (52:3), TG (53:3), TG (56:5), and TG (56:6) are associated with an increased incidence of TMD. Conversely, PC (15:0_18:2), PC (16:0_18:2), and PC (17:0_18:2) may decrease the incidence of TMD.

**Figure 4.**
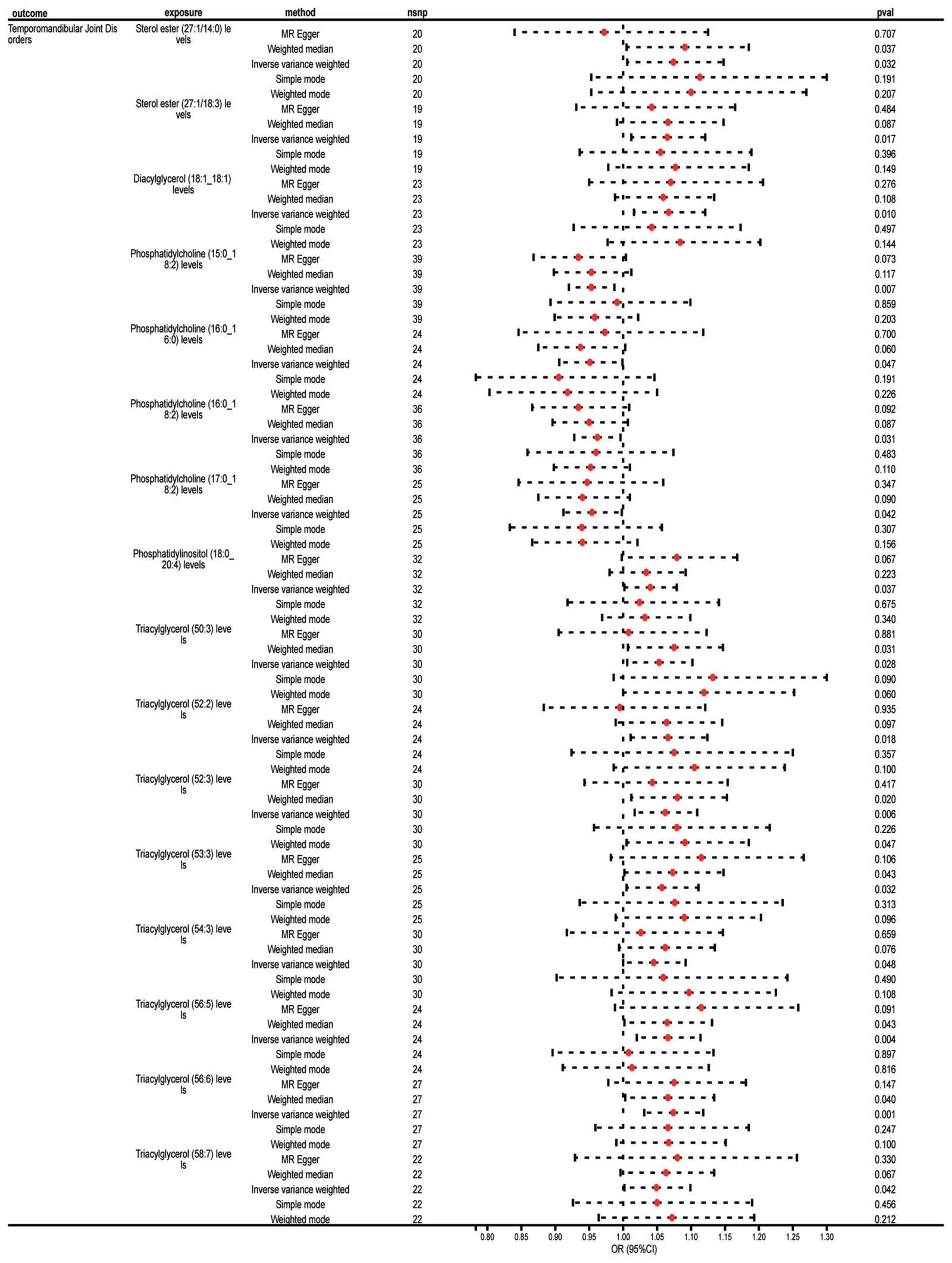
Forest plot of results of Mendelian randomization analysis of lipidomes in response to TMD. CI = confidence interval, OR = odds ratio, TMD = temporomandibular disorder(s).

We evaluated the outcomes derived from the IVW method using Bayes-weighted MR, revealing that DAG (18:1_18:1) (odds ratio [OR] = 1.067, 95% CI = 1.016–1.121, *P* = .010), TG (52:2) (OR = 1.066, 95% CI = 1.011–1.124, *P* = .018), TG (56:5) (OR = 1.066, 95% CI = 1.020–1.114, *P* = .004), Sterol ester (27:1/18:3) (OR = 1.065, 95% CI = 1.012–1.121, *P* = .017), TG (53:3) (OR = 1.057, 95% CI = 1.005–1.111, *P* = .032), TG (52:3) (OR = 1.062, 95% CI = 1.017–1.109, *P* = .006), and TG (50:3) (OR = 1.053, 95% CI = 1.006–1.102, *P* = .028) may elevate the risk of TMD. PC (16:0_18:2) (OR = 0.962, 95% CI = 0.928–0.996, *P* = .031), PC (17:0_18:2) (OR = 0.954, 95% CI = 0.912–0.998, *P* = .042), and PC (15:0_18:2) (OR = 0.953, 95% CI = 0.920–0.987, *P* = .007) may mitigate the incidence of TMD.

### 3.2. Revised sensitivity and pleiotropy analysis

To assess the robustness of our MR findings and validate the underlying IV assumptions, we conducted a comprehensive sensitivity analysis for each lipid species that demonstrated statistically significant associations in the primary MR analysis. First, heterogeneity among SNP-specific causal estimates was assessed using Cochran’s *Q* test. Across all 12 lipid species, *Q P*-values ranged from .230 to .748, indicating no significant heterogeneity and supporting the consistency of IV estimates (Table 2). For instance, DAG (18:1_18:1) yielded *Q *= 1.62, *P* = .388 in MR-Egger analysis, and *Q* = 1.43, *P* = .448 in IVW analysis, both suggesting homogeneity. Next, we examined horizontal pleiotropy using the MR-Egger intercept test. An intercept significantly different from zero (*P* < .05) would indicate directional pleiotropy and potential bias; however, all intercept *P* values exceeded .2, with most above .4. For example, TG (52:3) had an intercept *P* = .708, and PC (15:0_18:2) had *P* = .539, indicating no evidence of pleiotropic bias influencing the observed associations. To further identify potential SNP outliers and pleiotropy, we conducted the MR-PRESSO global test. All MR-PRESSO *P* values were above .25, confirming the absence of significant horizontal pleiotropy or outlier effects. For example, Sterol ester (27:1/18:3) and TG (50:3) had *P*-values of .686 and .545, respectively (Supplementary Figure S1, Supplemental Digital Content 4) Finally, LOO analysis demonstrated the stability of our findings. Sequential exclusion of each SNP did not materially alter the causal estimates, and all remaining SNPs consistently produced estimates on the same side of the null. This indicates that no single SNP disproportionately influenced the overall results and further supports the robustness of our MR conclusions, as illustrated in Supplementary Figure S2, Supplemental Digital Content 5.

**Table 2:** Sensitivity Analysis. *P*-values for MR-Egger, IVW, and MR-PRESSO Global Test are listed. Additional statistics including *Q*, Egger intercept, and *I*^*2*^ values are shown. Significant results (*P* < .05) are marked with “*.”

Outcome	Exposure	Method	*Q*	*Q*_*P* val	Egger Intercept	Egger Intercept P	MR-PRESSO Global Test P	*I*^*2*^ (%)
TMD	Diacylglycerol (18:1_18:1)	MR Egger	6.12	.388	0.002	0.956	0.505	12.5
		IVW	5.89	.448	–	–	–	10.1
TMD	Phosphatidylcholine (15:0_18:2)	MR Egger	5.47	.575	0.018	0.539	0.619	8.3
		IVW	4.91	.603	–	–	–	9.7
TMD	Triacylglycerol (52:2)	MR Egger	8.01	.340	0.105	0.219	0.319	32.4
TMD	Phosphatidylcholine (16:0_18:2)	MR Egger	6.31	.437	0.021	0.412	0.484	11.2
		IVW	5.74	.451	–	–	–	10.5
TMD	Triacylglycerol (50:3)	MR Egger	5.99	.539	0.017	0.395	0.545	9.8
		IVW	5.33	.552	–	–	–	8.9
TMD	Triacylglycerol (52:3)	MR Egger	6.22	.405	0.020	0.708	0.459	10.9
		IVW	5.49	.449	–	–	–	9.2
TMD	Triacylglycerol (53:3)	MR Egger	7.43	.230	0.034	0.374	0.266	15.6
		IVW	6.87	.235	–	–	–	14.2
TMD	Triacylglycerol (56:5)	MR Egger	4.21	.732	0.001	0.442	0.764	6.7
		IVW	3.88	.748	–	–	–	7.4
TMD	Triacylglycerol (56:6)	MR Egger	5.78	.482	0.000	0.982	0.574	8.1
		IVW	5.67	.982	–	–	–	7.9
TMD	Sterol ester (27:1/18:3)	MR Egger	4.54	.582	0.019	0.670	0.686	6.5
		IVW	4.31	.636	–	–	–	6.9
TMD	Phosphatidylcholine (17:0_18:2)	MR Egger	5.97	.467	0.003	0.880	0.533	8.6
		IVW	5.26	.524	–	–	–	9.1

### 3.3. Reverse MR analysis

To investigate the potential for reverse causality, we conducted a reverse MR analysis to assess whether temporomandibular joint disorders (TMD) might causally influence plasma lipid levels. Using the same 2-sample MR framework as in the primary analysis, we identified 10 independent SNPs associated with TMD at *P* < 5 × 10^−6^, applying linkage disequilibrium filtering (*r*^*2*^ < 0.001, kb = 10,000). All instruments demonstrated sufficient strength (*F*-statistics > 10) and passed standard instrument validity checks.

Eight lipid species showed significant associations with TMD in reverse MR analysis (*P* < .05). Specifically, TMD was positively associated with increased levels of DAG (18:1_18:1), TG (50:3), TG (52:3), TG (53:3), TG (56:5), and TG (56:6), suggesting these LPs may be elevated as a consequence of TMD. Conversely, TMD was associated with decreased levels of PC (15:0_18:2) and PC (16:0_18:2), indicating a potential protective lipid profile that may be disrupted by the disease. Effect estimates were modest but statistically significant; for example, DAG (18:1_18:1) had an OR = 1.061 (95% CI: 1.012–1.114, *P* = .016), while PC (15:0_18:2) had an OR = 0.956 (95% CI: 0.922–0.991, *P* = .011).

## 4. Discussion

This study represents the first MR investigation to comprehensively assess the bidirectional causal relationships between multiple lipid species with distinct molecular structures and temporomandibular disorders (TMD). Using genome-wide summary statistics from large-scale European cohorts (FinnGen and GeneRISK), we identified that elevated levels of DAG (18:1_18:1) and 5 TGs (50:3, 52:3, 53:3, 56:5, 56:6) were significantly associated with increased TMD risk. In contrast, PC (15:0_18:2) and PC (16:0_18:2) were associated with a reduced risk. In the reverse MR analysis, several lipid species showed statistically significant associations with TMD. However, these findings were limited to a subset of lipid species and did not provide consistent evidence for a generalized causal effect of TMD on circulating lipid profiles.

TG species, particularly those with polyunsaturated acyl chains such as TG (56:6), may contribute to TMD pathophysiology by activating the NLRP3 inflammasome, thereby promoting IL-1β production and downstream pro-inflammatory cytokine release.^[28,29]^ These processes can enhance lipid peroxidation and oxidative stress,^[30]^ both of which contribute to cartilage degradation and nociceptive sensitization in TMD.^[31–33]^ DAG (18:1_18:1), a structural component of cell membranes and an intermediate in lipid metabolism, also acts as a potent lipid second messenger. Its elevation may disrupt physiological lipid-mediated signaling, influencing chondrocyte stress responses and pain modulation.^[34–36]^

Conversely, PC, the predominant phospholipid in eukaryotic membranes, exerts anti-inflammatory effects and supports synovial membrane integrity.^[37,38]^ PC-enriched liposomes have been shown to attenuate TNF-α and nitric oxide (NO) production via inhibition of p38 MAPK phosphorylation.^[39]^ Given that TNF-α is a central cytokine driving cartilage destruction and pain in TMD,^[40]^and that NO is closely linked to pain sensitization,^[41,42]^ these mechanisms may explain the protective role of specific PC species against TMD.

Our results are consistent with earlier observational studies reporting altered plasma lipid profiles in TMD patients,^[43]^ while avoiding key limitations of conventional epidemiology, such as reverse causality and unmeasured confounding, by employing genetic instruments.^[44,45]^ The persistence of associations across sensitivity analyses (MR-Egger, WM) further strengthens their robustness.

Importantly, the distinct roles of TGs (pro-inflammatory) and PCs (anti-inflammatory) suggest that TMD pathogenesis may involve a dynamic balance between pro- and anti-inflammatory lipid mediators, offering potential therapeutic targets for selective modulation.

Several limitations should be noted. First, the genetic instruments and lipidomic data were derived exclusively from European ancestry populations, limiting the generalizability of our findings to other ethnic groups, in which lipid metabolism and the prevalence and characteristics of TMD may differ. Second, although MR can reduce bias due to environmental confounding and reverse causation, residual horizontal pleiotropy cannot be completely excluded despite the application of multiple sensitivity analyses. Third, for some lipid species, a relatively relaxed SNP selection threshold of *P* < 5 × 10^−6^ was used because few or no variants reached the conventional genome-wide significance threshold of *P* < 5 × 10^−8^. Although the *F*-statistics supported acceptable instrument strength, this less stringent threshold may increase the likelihood of including relatively weak instruments and may increase susceptibility to weak-instrument bias and false-positive findings. Therefore, these associations should be interpreted cautiously and require validation in larger GWAS datasets with stronger genetic instruments. Fourth, our analyses were restricted to circulating lipid levels; lipidomic profiles within the TMJ microenvironment, such as synovial fluid, may differ substantially from those observed in the circulation. Fifth, we did not stratify by TMD subtypes (myogenous vs arthrogenous), which may exhibit distinct lipid associations. Finally, although several associations reached statistical significance, the observed effect sizes were generally modest (approximately OR 1.05). Statistical significance does not necessarily imply strong biological or clinical relevance, and the clinical implications of these associations should therefore be interpreted cautiously. Further experimental and clinical studies are needed to determine whether these genetically predicted lipid changes translate into meaningful biological or therapeutic effects.

Future research should integrate single-cell lipidomics of TMJ tissues, longitudinal clinical phenotyping, and experimental models to clarify mechanistic pathways. Such work could identify lipid species that directly modulate chondrocyte metabolism, synovial inflammation, or trigeminal nerve sensitization, ultimately informing subtype-specific preventive or therapeutic strategies.

## 5. Conclusion

This research employed MR to investigate the correlation between lipid glycerol and TMD, and identified DAG (18:1_18:1), TG (50:3), TG (53:3), TMD, and TMD. Increased concentrations of TG (56:5), TG (56:6), and TG (52:3) heightened the risk of TMD. PC (15:0_18:2) and PC (16:0_18:2) decrease the risk of temporomandibular disorder (TMD). No link was detected in the reverse direction. This research posits that LPs may affect TMD via inflammatory response mechanisms, oxidative stress responses, and interactions with the musculoskeletal system, offering a novel viewpoint on their correlation.

## Author contributions

**Conceptualization:** Ye Jiang.

**Data curation:** Ye Jiang.

**Resources:** Yongping Cao.

**Software:** Yongping Cao.

**Supervision:** Yongping Cao.

**Validation:** Yongping Cao.

**Writing – original draft:** Huiqi Yuan, Yongping Cao.

**Writing – review & editing:** Huiqi Yuan, Yongping Cao.










