# Supplementary material for: Mendelian randomization of plasma lipidome and temporomandibular joint disorders
Source: Medicine (Baltimore). 2026 Oct 16;105(42):e50984. doi: 10.1097/MD.0000000000050984 (PMC13680941; doi:10.1097/MD.0000000000050984)
Supplement: Supplementary file 2 [file medi-105-e50984-s005.docx]

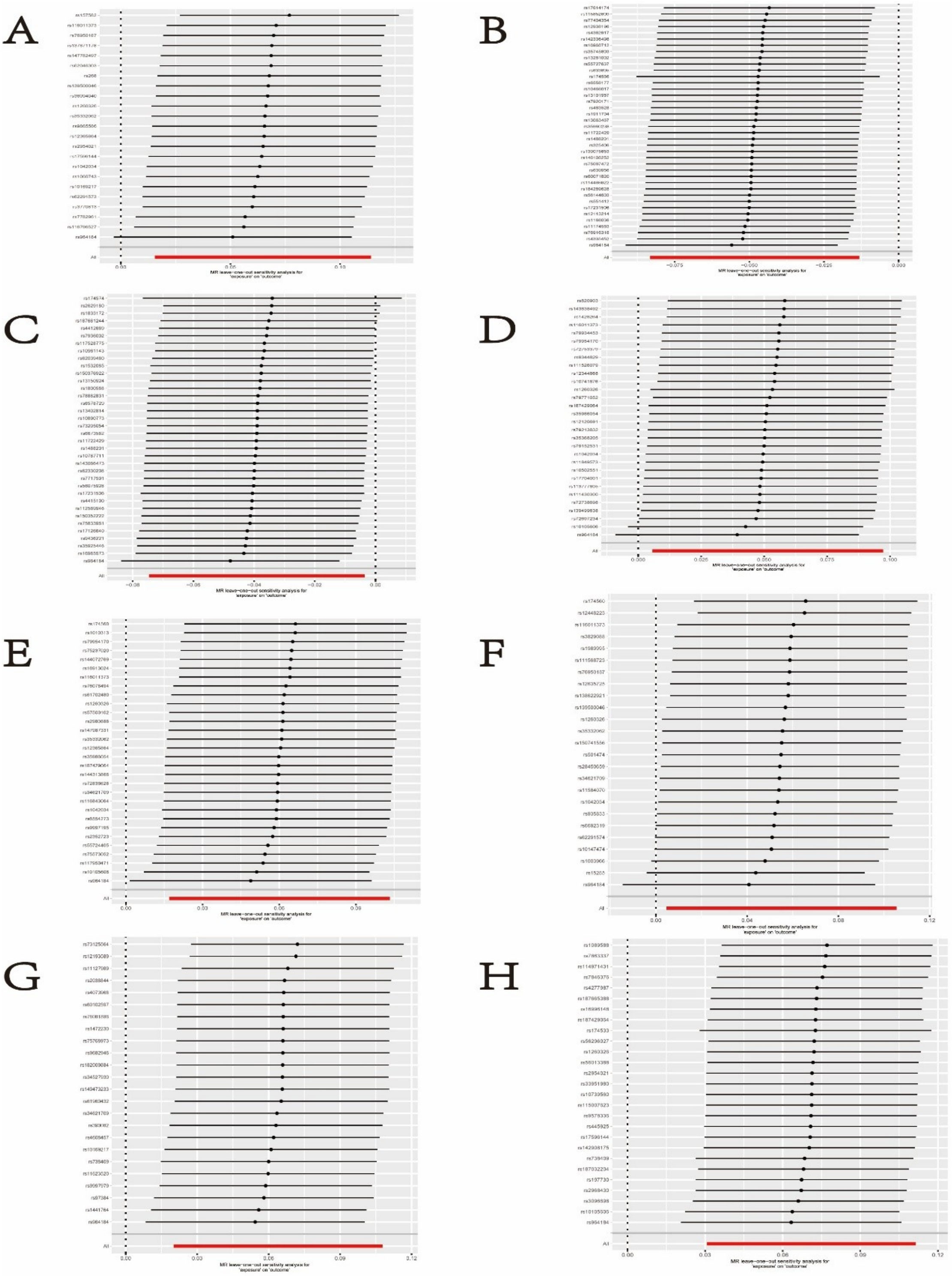
A
B
C
D
E
F
G
H
All
MR leave-one-out sensitivity analysis for 'exposure' on 'outcome'
